# Vision–Radar Semantic Communication for Target-Oriented Visual Sensing: Image Reconstruction and Dynamic State Estimation

**DOI:** 10.3390/s26185900

**Published:** 2026-09-17

**Authors:** Hao Xu, Yi Wang, Rikang Zhao, Qiang Liu

**Affiliations:** 1 Yangtze Delta Region Institute (Quzhou), University of Electronic Science and Technology of China, Quzhou 324003, China; wangyi@csj.uestc.edu.cn (Y.W.); liuqiang@uestc.edu.cn (Q.L.); 2 School of Information and Communication Engineering, University of Electronic Science and Technology of China, Chengdu 611731, China; 3 College of Electronic and Information Engineering, Shandong University of Science and Technology, Qingdao 266590, China

**Keywords:** sensing image processing, vision–radar sensing, semantic communication, target image reconstruction, dynamic state estimation

## Abstract

Target-oriented sensing image processing and analysis are important for intelligent surveillance and dynamic sensing applications, where wireless systems need to recover task-relevant visual and state information from heterogeneous sensing data. This study develops a vision–radar semantic communication system for joint target image reconstruction and dynamic state estimation. The transmitter jointly encodes scene-level visual observations and target-associated radar echoes through vision–radar semantic extraction and fusion, lightweight contextual semantic encoding, and communication-aware semantic transmission. The receiver uses a multi-task semantic decoder to reconstruct the target image and estimate normalized distance, angle, and velocity. Experiments are conducted under adaptive signal-to-noise ratio (SNR)–modulation operating points and evaluated using peak signal-to-noise ratio (PSNR), structural similarity index measure (SSIM), and normalized state-estimation root mean square errors (RMSEs). The developed system achieves an average PSNR of 24.57 dB, SSIM of 0.8896, Normalized Distance RMSE of 0.0120, Normalized Angle RMSE of 0.0059, and Normalized Velocity RMSE of 0.0015, outperforming a continuous-channel training variant on all reported average metrics. Ablation studies show that the composite reconstruction objective and adaptive task-balancing scheme provide favorable visual-sensing tradeoffs. These results indicate that vision–radar semantic communication can support target-oriented visual sensing and dynamic state recovery within a unified sensing-oriented wireless framework.

## 1. Introduction

Sensing image processing and analysis are essential for applications in which visual sensors observe targets or scenes from a distance, such as intelligent surveillance, traffic monitoring, and mobile perception [1,2,3]. In these scenarios, the receiver often requires target-level visual information together with dynamic state information for subsequent decision making [4]. Therefore, the sensing pipeline should support not only visual reconstruction but also target-oriented analysis under practical communication constraints.

Semantic communication has emerged as a promising paradigm for future wireless systems by shifting the communication objective from bit-level data recovery to task-relevant information delivery [5,6,7]. Instead of transmitting raw source data with strict fidelity requirements, semantic communication aims to extract, transmit, and recover compact semantic representations that are directly useful for receiver-side tasks [8,9]. Recent task-oriented and image-oriented semantic communication studies have further investigated semantic feature extraction, wireless image transmission, and receiver-side visual recovery under communication constraints [10,11]. These studies show that semantic communication is moving from source-level reconstruction toward task-aware information delivery, but they mainly focus on visual data transmission or visual semantic recovery.

Recent studies have also extended semantic communication to multimodal and multi-task settings [12,13,14]. These studies demonstrate that multiple modalities or multiple receiver-side tasks can be jointly considered to improve semantic efficiency under wireless constraints. However, their task settings are generally different from target-oriented vision–radar sensing, where the receiver needs to recover both a target image and normalized dynamic state variables after semantic transmission.

Dynamic target sensing often requires information from both vision and radar modalities [15,16]. Vision observations provide rich spatial structure and appearance cues, which are useful for target image reconstruction [17]. Radar echoes provide complementary sensing information associated with target distance, angle, and motion [18,19]. Recent radar–camera fusion studies further show that radar and visual observations can provide complementary information for robust target perception in traffic scenes [20,21]. However, these perception-oriented methods mainly focus on detection, localization, tracking, or segmentation, and usually assume local multimodal processing rather than semantic transmission over wireless links.

Therefore, a clear research gap remains for target-oriented visual sensing over wireless semantic communication links. Existing image-oriented semantic communication methods mainly focus on visual data transmission, reconstruction, or visual inference, but they generally do not jointly recover target images and dynamic target states [10,11]. Existing multi-task semantic communication methods consider multiple receiver-side tasks, but they do not specifically address vision–radar semantic fusion for joint target image reconstruction and normalized distance, angle, and velocity estimation [13,14]. Existing radar–camera perception methods exploit visual and radar complementarity for perception tasks, but they do not directly consider communication-aware semantic encoding and receiver-side multi-task recovery after wireless transmission [20,21]. As a result, the joint design of vision–radar semantic fusion, communication-aware transmission, and multi-task decoding for simultaneous target image reconstruction and normalized dynamic state estimation remains insufficiently explored.

To address this gap, this paper develops a vision–radar semantic communication system for target-oriented visual sensing, with joint target image reconstruction and dynamic state estimation as the receiver-side objectives. In the developed system, the transmitter jointly processes a scene-level visual observation and a target-associated radar echo to generate multimodal semantic representations. These representations are further encoded by a lightweight contextual semantic encoder and transmitted through a communication-aware semantic transmission module. At the receiver, a multi-task semantic decoder reconstructs the target image and estimates the normalized distance, angle, and velocity of the target. In this way, the developed system differs from image-only semantic communication, single-task sensing estimation, and perception-only vision–radar fusion by jointly considering vision–radar semantic transmission, target image reconstruction, and normalized target-state recovery within a unified sensing-oriented wireless framework.

The main contributions of this paper are summarized as follows.

- We develop a vision–radar semantic communication system for target-oriented visual sensing, where scene-level visual observations and target-associated radar echoes are jointly encoded at the transmitter, and the receiver reconstructs the target image while estimating normalized distance, angle, and velocity.
- We design an end-to-end semantic transmission pipeline that integrates vision–radar semantic extraction and fusion, lightweight contextual semantic encoding, communication-aware semantic transmission, and multi-task semantic decoding for joint target image reconstruction and normalized dynamic state estimation.
- We evaluate the developed system under adaptive signal-to-noise ratio (SNR)–modulation operating points using peak signal-to-noise ratio (PSNR), structural similarity index measure (SSIM), and normalized state-estimation root mean square errors (RMSEs). The results demonstrate improved target image reconstruction and normalized target-state estimation, while studies on reconstruction objectives and task-balancing schemes analyze the visual-sensing tradeoff.

The remainder of this paper is organized as follows. Section 2 reviews related work on sensing image processing, semantic communication, and vision–radar multimodal sensing. Section 3 presents the developed vision–radar semantic communication system. Section 4 describes the training objective and experimental setup. Section 5 reports the experimental results and ablation studies. Section 6 concludes the paper.

## 2. Related Work

### 2.1. Sensing Image Processing and Target-Oriented Analysis

Image processing and analysis have become fundamental components of modern sensing applications because visual observations must be transformed into task-level information for perception, monitoring, and decision making. In remote sensing and visual sensing research, deep learning has been widely studied for extracting semantic information from image data and supporting scene understanding, target recognition, and image-level analysis [22,23]. These studies show that learning-based image processing can provide powerful representations for sensing applications.

For dynamic target-oriented sensing, visual information alone is often insufficient because the receiver may need both appearance-related information and state-related information. Target image reconstruction focuses on preserving visual semantics such as shape, color, and structure, while target-state estimation focuses on distance, angle, and motion-related cues. Existing image processing studies mainly address image-level representation, recognition, segmentation, or reconstruction, but they do not directly consider how visual information should be jointly transmitted with radar-related sensing information under wireless communication constraints. Therefore, target-oriented visual sensing requires a framework that can preserve visual appearance information while supporting state-related recovery at the receiver.

### 2.2. Semantic Communication and Task-Oriented Wireless Transmission

Semantic communication has attracted increasing attention as a promising paradigm for future wireless networks. Different from conventional bit-level transmission, semantic communication focuses on delivering task-relevant information that is useful for receiver-side objectives. Early deep joint source-channel coding studies demonstrated that neural encoders and decoders can directly map source data into channel-input representations for wireless image transmission [24]. Recent task-oriented semantic communication further extends this idea by optimizing transmitted representations for downstream perception, inference, or reconstruction tasks [10,25]. Image-oriented semantic communication studies further demonstrate that task-aware visual semantics can improve wireless image transmission and receiver-side visual recovery [10,11].

Although these studies show the potential of semantic communication in reducing transmission redundancy and improving task-level efficiency, many of them focus on image data or a single receiver-side objective. Recent multimodal and multi-task semantic communication studies have started to consider multiple data sources or multiple receiver-side tasks [12,13,14]. However, the joint recovery of target images and normalized dynamic states remains different from general multi-task semantic recovery. In this setting, the transmitted representation should preserve both appearance-related visual semantics and radar-related state cues, and the training objective should balance visual reconstruction quality with distance, angle, and velocity estimation accuracy.

### 2.3. Vision–Radar Multimodal Sensing and Communication-Aware Transmission

Vision and radar modalities provide complementary information for dynamic target perception. Visual observations contain rich spatial, structural, and appearance cues, while radar echoes provide target-related information associated with distance, angle, and motion. By jointly exploiting visual and radar modalities, a sensing system can form more informative representations for target-oriented perception tasks. Representative radar–camera fusion methods have been studied for vehicle detection, 3D perception, localization, and robust perception under challenging conditions [16,18,20,21].

However, most radar–camera perception methods assume that visual and radar data are processed locally and do not explicitly model semantic transmission over wireless links. As a result, they do not address how vision–radar semantic representations can be encoded, transmitted, and decoded to support joint target image reconstruction and state estimation at a remote receiver. Recent work has explored semantic communication for multimodal sensing, showing that radar and visual information can be encoded into task-relevant semantic representations for target-oriented reconstruction and motion-parameter estimation [26,27]. These studies motivate the use of compact vision–radar semantics for sensing-oriented wireless applications, but the joint design of communication-aware semantic transmission, receiver-side target image reconstruction, normalized dynamic state estimation, and task balancing remains insufficiently analyzed.

Wireless semantic communication should also consider the effect of the communication process on learned semantic representations. In many end-to-end transmission systems, semantic features are optimized together with channel models so that the receiver can recover task-relevant information after wireless propagation. Recent studies have considered finite-alphabet or digital-compatible semantic transmission, including constellation-constrained deep joint source-channel coding [28] and digital semantic joint coding-modulation [29]. For target-oriented visual sensing, this issue is more complex because the same transmitted semantic representation must support both visual reconstruction and multiple normalized state-estimation outputs.

### 2.4. Comparative Summary and Research Gap

Based on the studies discussed above, Table 1 summarizes representative related study categories from the perspectives of input modality, semantic transmission, target image reconstruction, target-state estimation, and channel modeling.

As shown in Table 1, image semantic communication mainly focuses on visual transmission or reconstruction, but does not explicitly recover radar-related target states. Multimodal semantic communication provides useful foundations for semantic transmission with multiple data sources, but it is not specifically formulated for joint target image reconstruction and normalized distance, angle, and velocity estimation. Multi-task semantic communication considers task-oriented optimization, but its target tasks are generally different from the joint visual-state recovery problem considered in this work. Radar–camera perception methods exploit visual and radar complementarity for target detection, localization, or state estimation, but they generally do not consider wireless semantic transmission, channel modeling, or receiver-side target image reconstruction.

Therefore, the joint optimization of target image reconstruction and normalized dynamic state estimation under communication-aware vision–radar semantic transmission remains insufficiently explored. To address this gap, the developed system jointly encodes visual and radar semantics at the transmitter, transmits the fused representation through a communication-aware semantic transmission module, and decodes it at the receiver for both target image reconstruction and normalized target-state estimation.

## 3. Developed Vision–Radar Semantic Communication System

### 3.1. System Overview

**Figure 1:**
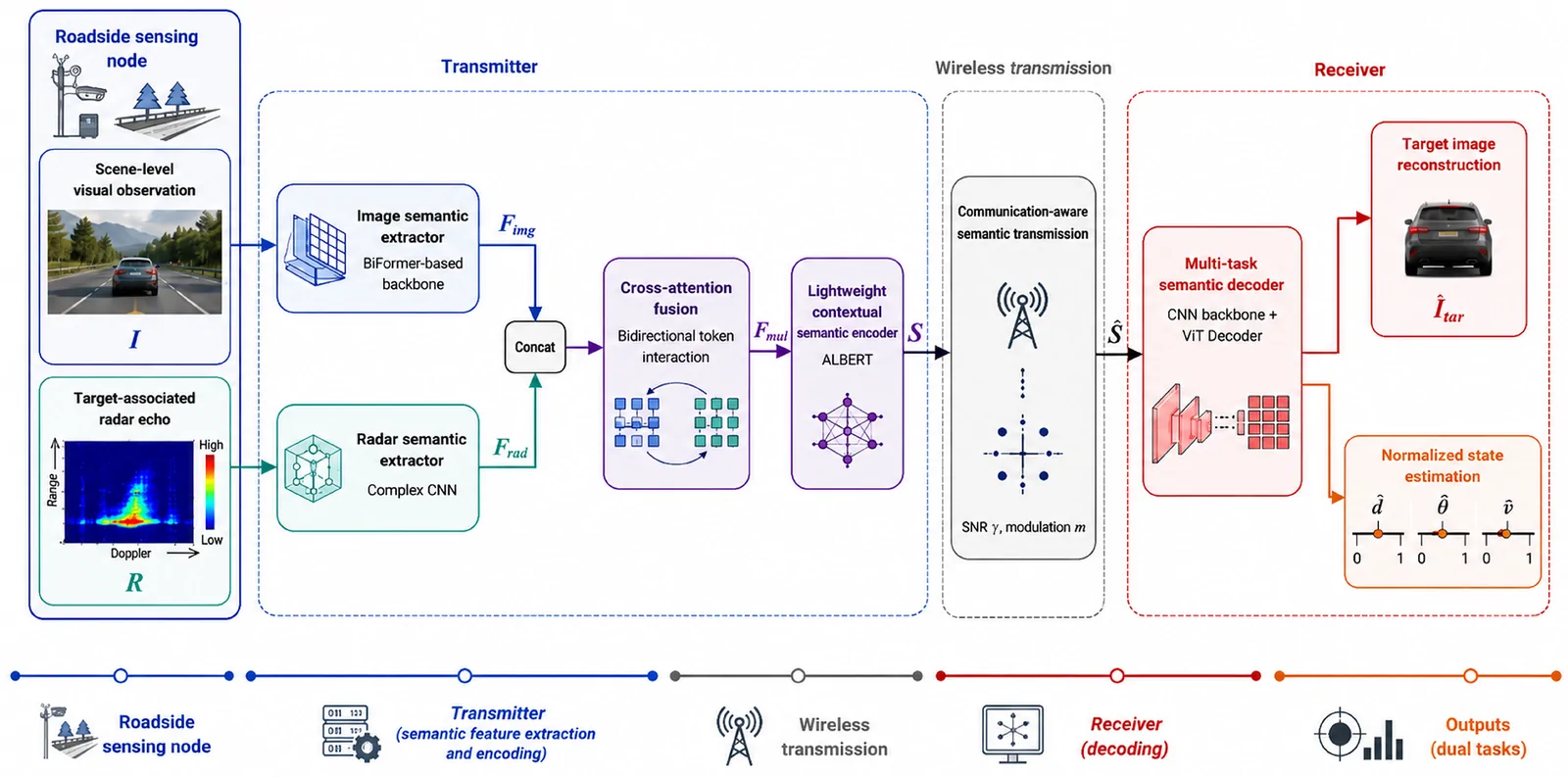
Overview of the developed vision–radar semantic communication system for joint target image reconstruction and dynamic state estimation. The transmitter extracts and fuses visual and radar semantic features, the communication module transmits the semantic representation under adaptive wireless operating points, and the receiver reconstructs the target image and estimates normalized distance, angle, and velocity.

This paper develops a vision–radar semantic communication system for joint target image reconstruction and dynamic state estimation, as shown in Figure 1. The system consists of transmitter-side semantic processing, communication-aware semantic transmission, and receiver-side target recovery.

At the transmitter, a scene-level visual observation and a target-associated radar echo are used as two sensing inputs. The image semantic extractor extracts appearance-related visual features from the scene-level visual observation, while the radar semantic extractor extracts target-state-related sensing features from the radar echo. These modality-specific features are projected into a shared semantic space and fused by the cross-attention fusion module to obtain multimodal semantic tokens.

The fused multimodal semantic tokens are further processed by the lightweight contextual semantic encoder to model token-level contextual relationships. The resulting semantic representation is then passed to the communication-aware semantic transmission module, where it is mapped into a transmission-compatible representation and propagated under the selected signal-to-noise ratio (SNR) and modulation setting.

At the receiver, the received semantic representation is decoded by the multi-task semantic decoder. The decoder contains a target-image reconstruction branch and a target-state estimation branch. The reconstruction branch recovers the target image, while the state-estimation branch predicts the normalized distance, angle, and velocity. In Figure 1, these steps correspond to the main blocks from semantic extraction, cross-attention fusion, contextual semantic encoding, wireless semantic transmission, and multi-task decoding. In this way, the developed system connects transmitter-side vision–radar semantic fusion with receiver-side joint target image reconstruction and normalized dynamic state estimation.

In the implementation, the image semantic extractor employs a BiFormer-tiny visual backbone and maps the final visual feature map into 49 visual semantic tokens with a 512-dimensional token representation. The radar semantic extractor contains three complex-valued one-dimensional residual convolutional blocks with channel dimensions of 10–128–256–512, and the extracted radar features are projected into 49 radar semantic tokens. The visual and radar semantic tokens are fused by a cross-attention module with a 512-dimensional token representation and 8 attention heads. The fused tokens are then mapped to the 128-dimensional input embedding space of the frozen ALBERT encoder, whose contextual semantic representation has a hidden dimension of 768. At the receiver, the multi-task decoder selects the 49 semantic tokens, reshapes them into a 7 × 7 feature map, and uses CNN-based feature refinement, a ViT decoder, and three scalar regression heads for target image reconstruction and normalized distance, angle, and velocity estimation.

### 3.2. Vision–Radar Semantic Extraction and Fusion

The transmitter contains two modality-specific semantic extraction branches. The image semantic extractor processes the scene-level visual observation and extracts visual features related to target appearance, spatial structure, and local scene context. In the implementation, the image branch is built on a BiFormer-based visual backbone [30]. The extracted visual feature map is converted into a sequence of visual semantic tokens before multimodal fusion.

The radar semantic extractor processes the target-associated radar echo and extracts sensing features related to target distance, angle, and motion. In the implementation, the radar branch uses a lightweight complex-valued convolutional neural network (CNN). Complex convolutional residual blocks are used to extract radar signal features, and the real and imaginary components are combined to form radar semantic tokens. This design keeps the radar branch compact while preserving target-state-related sensing cues.

The modality-specific semantic extraction process is expressed as:

(1) $$F_{\mathrm{img}}=f_{\mathrm{BiF}}\left(I\right),$$

(2) $$F_{\mathrm{rad}}=f_{\mathrm{cCNN}}\left(R\right),$$

where I denotes the scene-level visual observation, R denotes the target-associated radar echo, $f_{\mathrm{BiF}}\left(\cdot \right)$ denotes the BiFormer-based image semantic extractor, and $f_{\mathrm{cCNN}}\left(\cdot \right)$ denotes the complex-valued CNN-based radar semantic extractor. The two outputs are represented as token sequences with a common semantic dimension, so that visual appearance cues and radar-derived state cues can be fused at the token level.

For notation convenience, the visual and radar token sequences are written as a paired multimodal token set:

(3) $$F_{\mathrm{cat}}=\mathrm{Concat}\left(F_{\mathrm{img}},F_{\mathrm{rad}}\right),$$

where Concat(·) denotes the concatenation of the two modality-specific token sequences along the token dimension. The fused multimodal semantic representation is then obtained by

(4) $$F_{\mathrm{mul}}=f_{\mathrm{CA}}\left(F_{\mathrm{cat}}\right),$$

where $f_{\mathrm{CA}}\left(\cdot \right)$ denotes the cross-attention-based vision–radar fusion module and $F_{\mathrm{mul}}$ denotes the fused multimodal semantic tokens.

In the implementation, the cross-attention fusion module models bidirectional token interaction between visual and radar semantic tokens. Through this process, radar-derived state cues interact with visual appearance information before semantic transmission, and the resulting representation is used as the input to the lightweight contextual semantic encoder.

### 3.3. Lightweight Contextual Semantic Encoding

After vision–radar semantic fusion, the fused multimodal semantic tokens are further processed by a lightweight contextual semantic encoder. This module models token-level contextual relationships among the fused semantic tokens and produces the semantic representation for communication-aware transmission.

In the developed system, the contextual semantic encoder is implemented using ALBERT [31]. ALBERT adopts factorized embedding parameterization and cross-layer parameter sharing, which enables contextual token modeling with a relatively compact parameter scale. In our implementation, the encoder contains 12 Transformer encoder layers, 12 attention heads, a hidden size of 768, and an embedding size of 128. The number of parameters of the contextual encoder is approximately 11.7 M. The encoder is initialized from the ALBERT pre-trained model, which was pre-trained on large-scale text corpora including English Wikipedia and BookCorpus.

The selected wireless operating point is introduced to the contextual encoder through channel-condition text tokens. Let $E_{\gamma ,m}$ denote the embedding associated with the SNR γ and modulation setting *m*. The contextual encoder input is written as

(5) $$X_{\mathrm{ctx}}=\mathrm{Concat}\left(F_{\mathrm{mul}},E_{\gamma ,m}\right),$$

where Concat(·) denotes token-wise concatenation. The contextual semantic representation is then given by

(6) $$S=f_{\mathrm{ALBERT}}\left(X_{\mathrm{ctx}}\right),$$

where $f_{\mathrm{ALBERT}}\left(\cdot \right)$ denotes the ALBERT-based contextual semantic encoder. The resulting semantic representation S is passed to the communication-aware semantic transmission module.

The parameters of the contextual semantic encoder are frozen during training. This setting reduces the number of trainable parameters and provides a stable contextual mapping for the fused vision–radar semantic tokens. In the joint optimization of target image reconstruction and state estimation, a fixed contextual encoder helps avoid excessive drift of the semantic token space while allowing the surrounding trainable modules to adapt to the task objectives and wireless operating points.

It should be noted that freezing the contextual semantic encoder does not freeze the whole transmitter. The image semantic extractor, radar semantic extractor, feature projection, cross-attention fusion module, communication-aware semantic transmission module, and multi-task semantic decoder remain trainable. Therefore, the vision–radar fusion and receiver-side recovery process can still adapt the transmitted semantic representation to the target-oriented tasks and adaptive wireless operating points.

### 3.4. Communication-Aware Semantic Transmission

The semantic representation is transmitted through a communication-aware semantic transmission module. This module maps the learned semantic representation into a wireless-transmission-compatible form, propagates it through the channel, and provides the received semantic representation for receiver-side decoding.

Under an SNR condition γ and modulation setting *m*, the received semantic representation is written as

(7) $$\hat{S}=T_{\mathrm{ch}}\left(S;\gamma ,m\right).$$

Here, $T_{\mathrm{ch}}\left(\cdot \right)$ represents the combined effect of semantic-to-channel mapping, modulation-dependent transmission, channel propagation, and receiver-side semantic recovery.

The communication module is incorporated into the end-to-end learning pipeline, so that the semantic representation can be optimized together with the receiver-side visual reconstruction and state-estimation tasks. Since $T_{\mathrm{ch}}\left(\cdot \right)$ depends on the SNR condition γ and modulation setting *m*, the trainable semantic extraction, fusion, transmission, and decoding modules are optimized under the same adaptive wireless operating points used in evaluation.

### 3.5. Multi-Task Semantic Decoder

At the receiver side shown in Figure 1, the received semantic representation $\hat{S}$ is decoded by a multi-task semantic decoder. The decoder is designed to recover both target visual information and normalized target-state information from the same received semantic representation.

In the implementation, the decoder first extracts the semantic tokens corresponding to the fused vision–radar representation from the received sequence. These tokens are organized as a compact spatial feature map and processed by a lightweight CNN backbone to obtain a receiver-side latent representation:

(8) $$Z=f_{\mathrm{CNN}}\left(\hat{S}\right),$$

where $f_{\mathrm{CNN}}\left(\cdot \right)$ denotes the receiver-side token-to-feature mapping and convolutional feature extraction process. This representation provides the shared semantic basis for the following target reconstruction and state-estimation branches.

For visual recovery, the reconstruction branch uses a ViT decoder [32] to reconstruct the target image:

(9) $${\hat{I}}_{\mathrm{tar}}=f_{\mathrm{ViTDec}}\left(Z\right),$$

where $f_{\mathrm{ViTDec}}\left(\cdot \right)$ denotes the ViT reconstruction decoder. This branch uses Transformer decoder blocks to model relationships among visual tokens and recover the target image from the receiver-side latent representation.

For target-state estimation, a separate state-estimation branch applies a convolutional projection followed by three scalar regression heads. The normalized distance, angle, and velocity are predicted as:

(10) $$\hat{d}=f_{d}\left(Z\right),$$

(11) $$\hat{\theta }=f_{\theta }\left(Z\right),$$

(12) $$\hat{v}=f_{v}\left(Z\right),$$

where $f_{d}\left(\cdot \right)$, $f_{\theta }\left(\cdot \right)$, and $f_{v}\left(\cdot \right)$ denote the regression heads for normalized distance, angle, and velocity, respectively. The complete receiver output is therefore

(13) $$\hat{Y}=\left\{{\hat{I}}_{\mathrm{tar}},\hat{d},\hat{\theta },\hat{v}\right\}.$$

By using a shared receiver-side semantic representation before separating the reconstruction and state-estimation branches, the decoder supports joint target-oriented recovery while keeping the visual and state outputs explicitly separated.

## 4. Training Objective and Experimental Setup

### 4.1. Training Objective

The developed system is trained to jointly support target image reconstruction and normalized target-state estimation. For each target of interest, the receiver outputs a reconstructed target image and three normalized state estimates, including distance, angle, and velocity. The training objective therefore consists of a reconstruction objective and three state-estimation objectives.

For target image reconstruction, the reconstruction objective combines mean squared error (MSE), $L_{1}$ loss, and structural similarity index measure (SSIM)-related terms, and is defined as

(14) $$L_{\mathrm{rec}}=\lambda_{\mathrm{mse}}L_{\mathrm{mse}}+\lambda_{l1}L_{l1}+\lambda_{\mathrm{ssim}}L_{\mathrm{ssim}},$$

where $L_{\mathrm{mse}}$, $L_{l1}$, and $L_{\mathrm{ssim}}$ denote the MSE, $L_{1}$, and SSIM-related loss terms, respectively. The corresponding coefficients are set to $\lambda_{\mathrm{mse}}=10.0$, $\lambda_{l1}=5.0$, and $\lambda_{\mathrm{ssim}}=1.0$. The MSE term measures pixel-wise reconstruction fidelity, the $L_{1}$ term provides an absolute-error constraint, and the SSIM-related term encourages structural consistency between the reconstructed target image and the ground-truth target image.

For target-state estimation, the three normalized sensing losses are defined as:

(15) $$L_{d}=\mathrm{MSE}\left(\hat{d},d\right),$$

(16) $$L_{\theta }=\mathrm{MSE}\left(\hat{\theta },\theta \right),$$

(17) $$L_{v}=\mathrm{MSE}\left(\hat{v},v\right),$$

where $L_{d}$, $L_{\theta }$, and $L_{v}$ correspond to normalized distance, angle, and velocity estimation, respectively.

To balance the reconstruction and state-estimation tasks, the developed system adopts an uncertainty-based adaptive task-balancing strategy. Let $L_{k}$ denote the loss of task *k*, where k∈{d,θ,v,rec}. A learnable task parameter $s_{k}$ is assigned to each task, and the total objective is written as

(18) $$L_{\mathrm{total}}=\sum_{k\in \{d,\theta ,v,\mathrm{rec}\}}\left(\exp\left(-s_{k}\right)L_{k}+s_{k}\right).$$

In this formulation, the effective task weights are learned as:

(19) $$\lambda_{d}^{\mathrm{eff}}=\exp\left(-s_{d}\right),$$

(20) $$\lambda_{\theta }^{\mathrm{eff}}=\exp\left(-s_{\theta }\right),$$

(21) $$\lambda_{v}^{\mathrm{eff}}=\exp\left(-s_{v}\right),$$

(22) $$\lambda_{\mathrm{rec}}^{\mathrm{eff}}=\exp\left(-s_{\mathrm{rec}}\right).$$

Thus, the sensing losses are not optimized with a fixed manual ratio throughout training. Instead, the relative contributions of target image reconstruction, normalized distance estimation, normalized angle estimation, and normalized velocity estimation are adjusted by learnable task parameters. This strategy is adopted because the reconstruction and state-estimation losses have different numerical scales and optimization behaviors. The adaptive formulation reduces manual weight tuning, prevents one task from dominating the shared semantic representation, and provides a more flexible tradeoff between visual reconstruction quality and dynamic state-estimation accuracy.

### 4.2. Evaluation Metrics

The visual reconstruction quality is evaluated using peak signal-to-noise ratio (PSNR) and structural similarity index measure (SSIM). PSNR measures pixel-level reconstruction fidelity, while SSIM evaluates structural similarity between the reconstructed target image and the ground-truth target image. For an evaluated image in the [0,1] range, PSNR is computed as

(23) $$\mathrm{PSNR}=10\log_{10}\left(\frac{1}{\mathrm{MSE}}\right),$$

where MSE denotes the mean squared error between the reconstructed target image and the ground-truth target image. SSIM is computed as

(24) $$\mathrm{SSIM}\left(x,y\right)=\frac{\left(2\mu_{x}\mu_{y}+C_{1}\right)\left(2\sigma_{xy}+C_{2}\right)}{\left(\mu_{x}^{2}+\mu_{y}^{2}+C_{1}\right)\left(\sigma_{x}^{2}+\sigma_{y}^{2}+C_{2}\right)},$$

where *x* and *y* denote the reconstructed and ground-truth target images, $\mu_{x}$ and $\mu_{y}$ are the mean intensities, $\sigma_{x}^{2}$ and $\sigma_{y}^{2}$ are the variances, $\sigma_{xy}$ is the covariance, and $C_{1}$ and $C_{2}$ are stability constants.

For normalized target-state estimation, the receiver is evaluated using normalized root mean square errors (RMSEs). Given *N* test samples, the Normalized Distance RMSE is computed as

(25) $${\mathrm{RMSE}}_{d}=\sqrt{\frac{1}{N}\sum_{i=1}^{N}{\left({\hat{d}}_{i}-d_{i}\right)}^{2}}.$$

Similarly, Normalized Angle RMSE and Normalized Velocity RMSE are computed as:

(26) $${\mathrm{RMSE}}_{\theta }=\sqrt{\frac{1}{N}\sum_{i=1}^{N}{\left({\hat{\theta }}_{i}-\theta_{i}\right)}^{2}},$$

(27) $${\mathrm{RMSE}}_{v}=\sqrt{\frac{1}{N}\sum_{i=1}^{N}{\left({\hat{v}}_{i}-v_{i}\right)}^{2}}.$$

Since distance, angle, and velocity labels are represented in normalized label space, the state-estimation metrics are reported as Normalized Distance RMSE, Normalized Angle RMSE, and Normalized Velocity RMSE.

### 4.3. Dataset Construction

The experiments are conducted on a target-oriented multimodal sensing dataset constructed from the Video and Image Retrieval and Analysis Tool (VIRAT) Video Dataset [33]. Four representative scenes are selected from the VIRAT videos. The scene selection considers the presence of moving vehicle targets, stable surveillance viewpoints, and sufficient target visibility, so that each selected scene can provide target-oriented visual samples for reconstruction and state estimation.

The VIRAT videos are sampled at one frame per second, resulting in approximately 14,000 scene-level visual observations. For each sampled frame, car targets are extracted as target-oriented reconstruction labels, producing approximately 79,000 target car images. Each data sample is organized around a target of interest. The transmitter-side input consists of the scene-level visual observation and a target-associated radar echo, while the receiver-side supervision consists of the corresponding target car image and normalized target-state labels, including distance, angle, and velocity. The distance, angle, and velocity labels are represented in the normalized range [0,1].

The target-associated radar echo is generated from the target-state information to provide a radar modality aligned with the visual target. For each target, the state tuple (r,θ,v) is used, where *r*, θ, and *v* denote the target distance, angle, and velocity, respectively. The radar signal is generated based on a simplified linear frequency-modulated target-return model. In this model, the distance determines the round-trip propagation delay, the velocity introduces a Doppler-related phase variation, and the angle determines the inter-channel phase response across the receiving antennas.

The echo received by the *p*-th antenna channel can be compactly written as

(28) $$R_{p}\left(t\right)=a_{p}\left(\theta \right)\beta \left(r\right)s\left(t-\tau \left(r\right)\right)\exp\left(j2\pi f_{D}\left(v\right)t\right)+n_{p}\left(t\right),$$

where s(t) denotes the transmitted linear frequency-modulated signal, τ(r)=2r/c is the distance-dependent round-trip delay, $f_{D}\left(v\right)$ denotes the Doppler-related term, $a_{p}\left(\theta \right)$ denotes the angle-dependent array response of the *p*-th receiving channel, β(r) denotes the distance-dependent reflection coefficient, and $n_{p}\left(t\right)$ denotes complex Gaussian noise.

This procedure embeds range-, angle-, and Doppler-related cues into the complex radar echo. The generated radar echo is then processed by the radar semantic extractor, rather than being directly used as a state label, so that the model learns state-related semantic representations from the radar input.

To further examine the robustness of the developed system, test-time radar perturbations are considered in the evaluation. The perturbations include additive complex Gaussian noise, random amplitude gain variation, and random phase rotation.

### 4.4. Adaptive Wireless Operating Points

The developed system is evaluated under predefined adaptive SNR–modulation operating points. For each evaluated SNR condition, the modulation format is selected according to the channel quality, so that lower-order modulation is used under low-SNR conditions and higher-order modulation is used under high-SNR conditions. Specifically, BPSK is used at 0 and 5 dB, QPSK is used at 10 and 15 dB, 8PSK is used at 20 dB, and 16-QAM is used at 25 dB.

For each operating point, the receiver performance is measured using both PSNR, SSIM, Normalized Distance RMSE, Normalized Angle RMSE, and Normalized Velocity RMSE. Throughout the experiments, the same adaptive wireless operating points are used for all compared system variants and ablation settings to ensure comparison under the same communication protocol.

### 4.5. Compared System Variants and Ablation Settings

The experimental evaluation considers the developed system, the continuous-channel training variant, modality-availability ablations, reconstruction-objective ablations, task-balancing ablations, and test-time radar perturbation settings.

For the transmission-setting comparison, the continuous-channel training variant uses the same architecture, dataset setting, reconstruction objective, task-balancing scheme, training length, and evaluation protocol as the developed system. The main difference is the training transmission process, so this variant is used to assess the influence of incorporating the communication process into training.

For modality-availability analysis, two single-modality variants are evaluated: vision-only semantic transmission and radar-only semantic transmission. The vision-only variant uses only the visual semantic branch, while the radar-only variant uses only the radar semantic branch. These variants are used to analyze the contribution of each sensing modality to target image reconstruction and normalized state estimation.

For the reconstruction-objective ablation, three settings are compared: MSE objective, MSE+L1 objective, and composite reconstruction objective. For the task-balancing ablation, four settings are compared: balanced fixed scheme, mild fixed scheme, state-aware fixed scheme, and adaptive balancing scheme. In addition, test-time radar perturbation evaluation is used to examine robustness under radar input variations. All variants and ablation settings are evaluated under the same adaptive wireless operating points.

### 4.6. Experimental Reporting Protocol

All main results are reported using the epoch-50 model to ensure a consistent comparison across system variants and ablation studies. The same adaptive wireless operating points and reporting metrics are used for all compared variants and ablation studies. The dataset is divided into training and test subsets with a ratio of 90% and 10%, respectively. The same data split is used for all compared variants and ablation studies, and the main training seed is fixed to 2048. The model is trained using the AdamW optimizer with a batch size of 48, a learning rate of $2\times 10^{-4}$, and a weight decay of $1\times 10^{-4}$. The main performance metrics include PSNR, SSIM, Normalized Distance RMSE, Normalized Angle RMSE, and Normalized Velocity RMSE.

## 5. Results and Discussion

### 5.1. Representative Target Image Reconstruction

**Figure 2:**
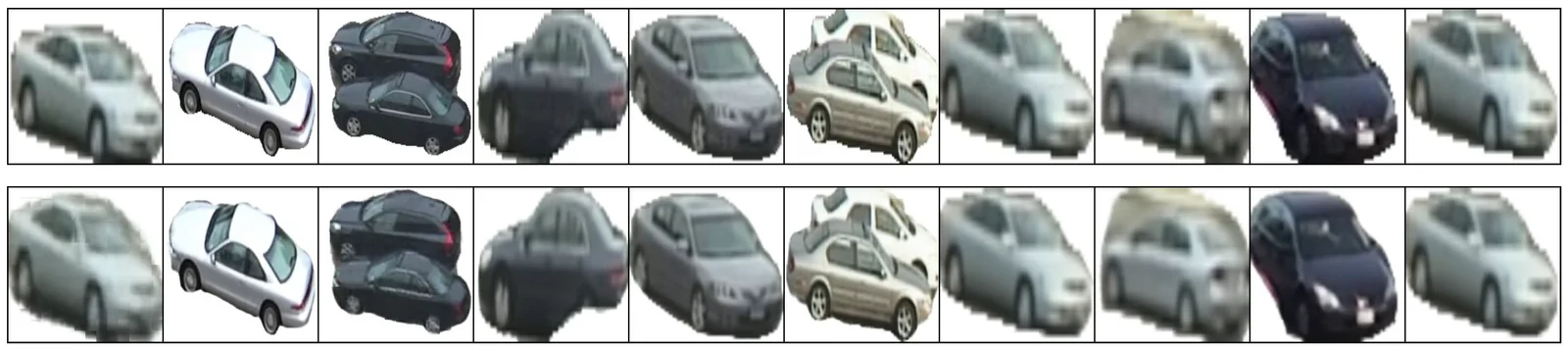
Representative target image reconstruction results. The top row shows the ground-truth target images, and the bottom row shows the corresponding reconstructed images.

Figure 2 presents representative target image reconstruction examples of the developed vision–radar semantic communication system. The top row shows the ground-truth target images, and the bottom row shows the corresponding reconstructed images. The reconstructed targets preserve the main vehicle contours, color characteristics, and target-level semantic appearance, demonstrating that the developed system can recover task-relevant visual information for target-oriented visual sensing.

### 5.2. Performance Comparison Under Adaptive Wireless Operating Points

This section evaluates the developed system and the continuous-channel training variant under the same adaptive wireless operating points. The comparison considers both target image reconstruction and normalized dynamic state estimation. Visual reconstruction is measured by PSNR and SSIM, while state-estimation accuracy is measured by Normalized Distance RMSE, Normalized Angle RMSE, and Normalized Velocity RMSE.

To assess the influence of the training transmission setting, the continuous-channel training variant is constructed with the same architecture, dataset setting, reconstruction objective, task-balancing scheme, training length, and evaluation protocol as the developed system. The main difference lies in the training transmission process. Table 2 reports the average performance of the two system variants.

As shown in Table 2, the developed system achieves higher average target reconstruction quality and lower normalized state-estimation errors than the continuous-channel training variant. Specifically, PSNR increases from 23.55 dB to 24.57 dB, and SSIM increases from 0.8670 to 0.8896. For target-state estimation, Normalized Distance RMSE, Normalized Angle RMSE, and Normalized Velocity RMSE are reduced from 0.0432, 0.0486, and 0.0028 to 0.0120, 0.0059, and 0.0015, respectively. These results indicate that incorporating the communication process during training improves the average visual-sensing performance of the receiver. The developed system was further evaluated using different training seeds while keeping the same data split and evaluation protocol. Over the repeated runs, the standard deviations of PSNR, SSIM, Normalized Distance RMSE, Normalized Angle RMSE, and Normalized Velocity RMSE were 0.06 dB, 0.0021, 0.0013, 0.0005, and 0.0001, respectively. These repeated-run results show small variations and consistent performance trends, supporting the stability of the developed system under different training seeds.

**Figure 3:**
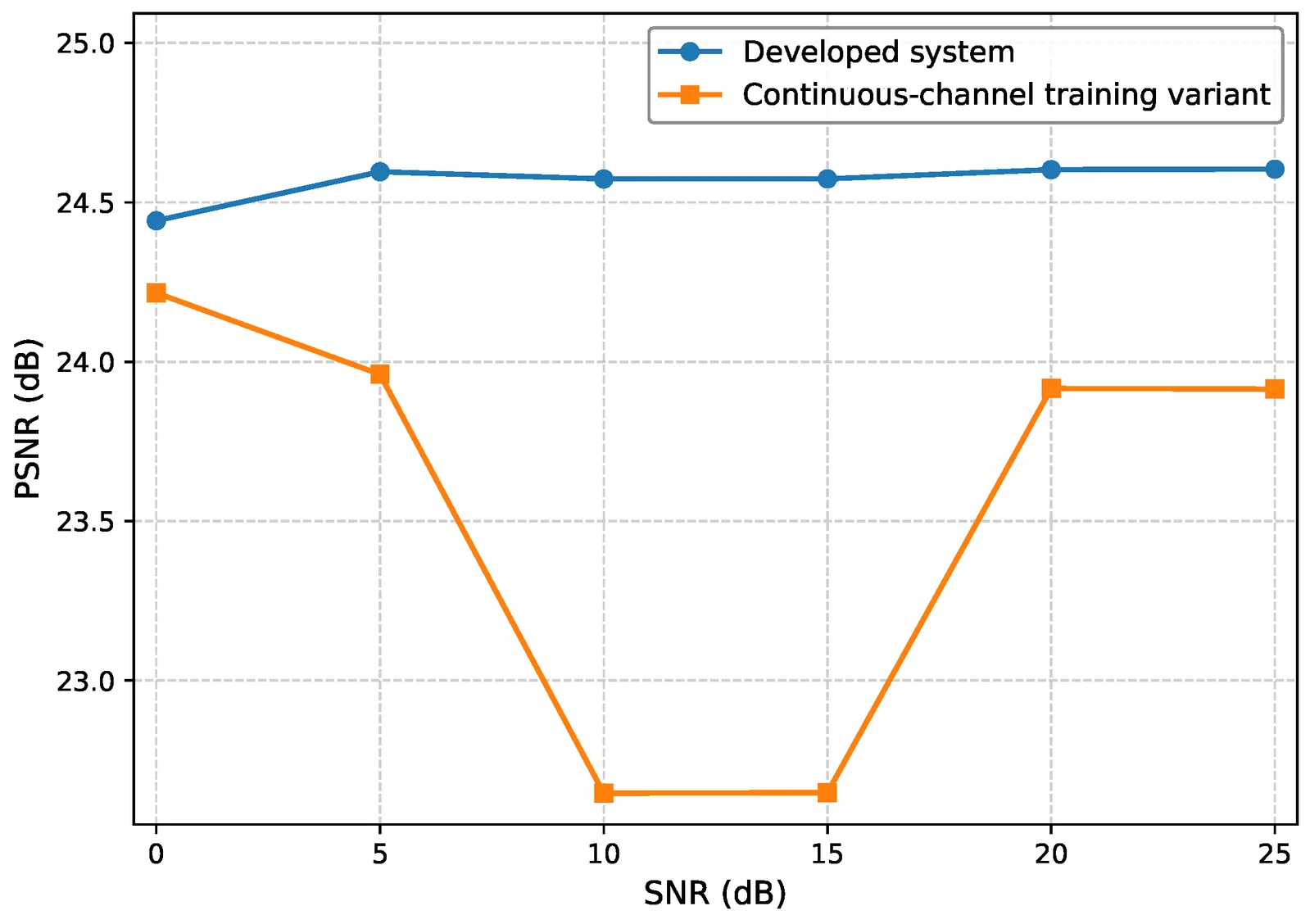
PSNR under adaptive wireless operating points.

**Figure 4:**
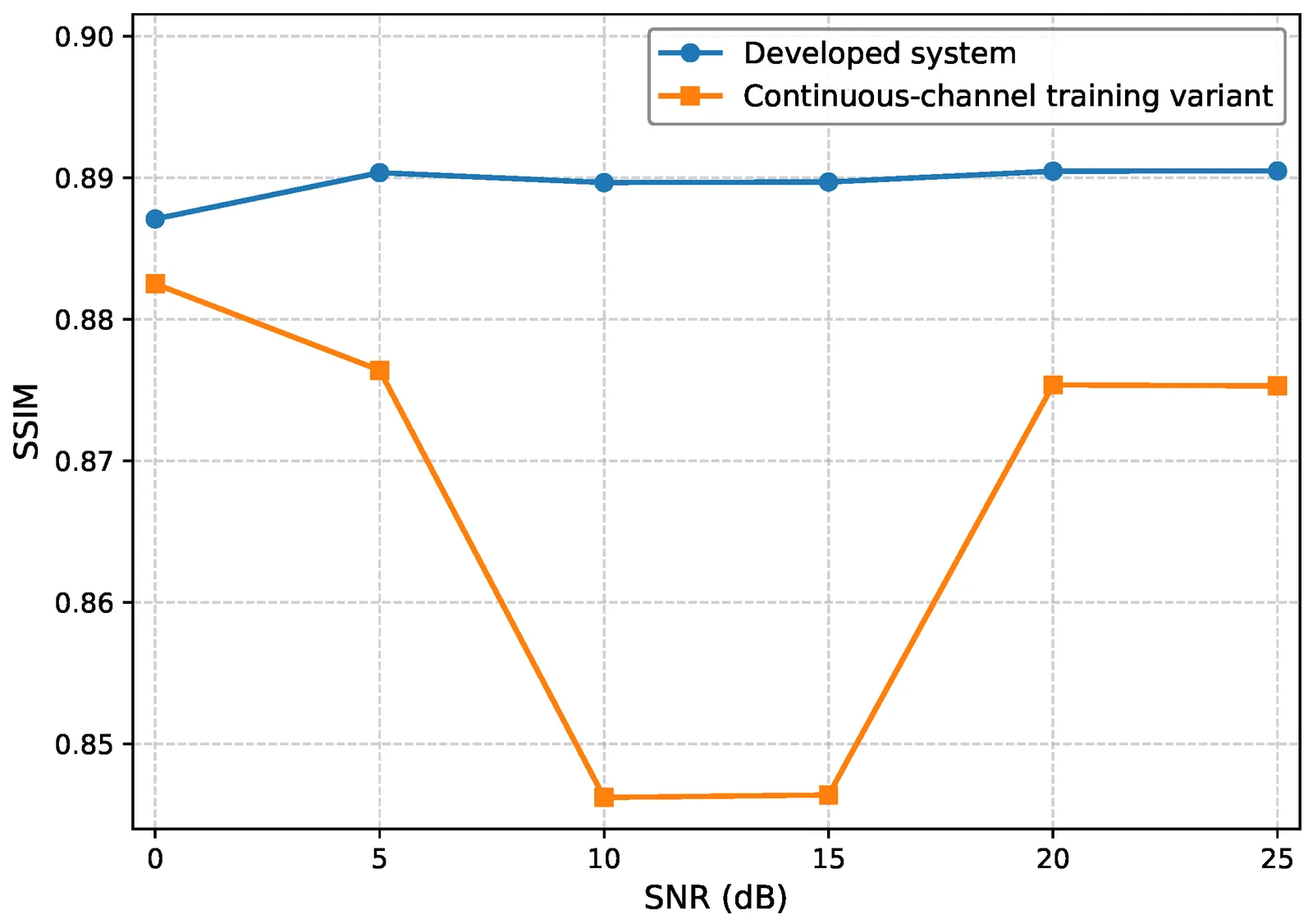
SSIM under adaptive wireless operating points.

**Figure 5:**
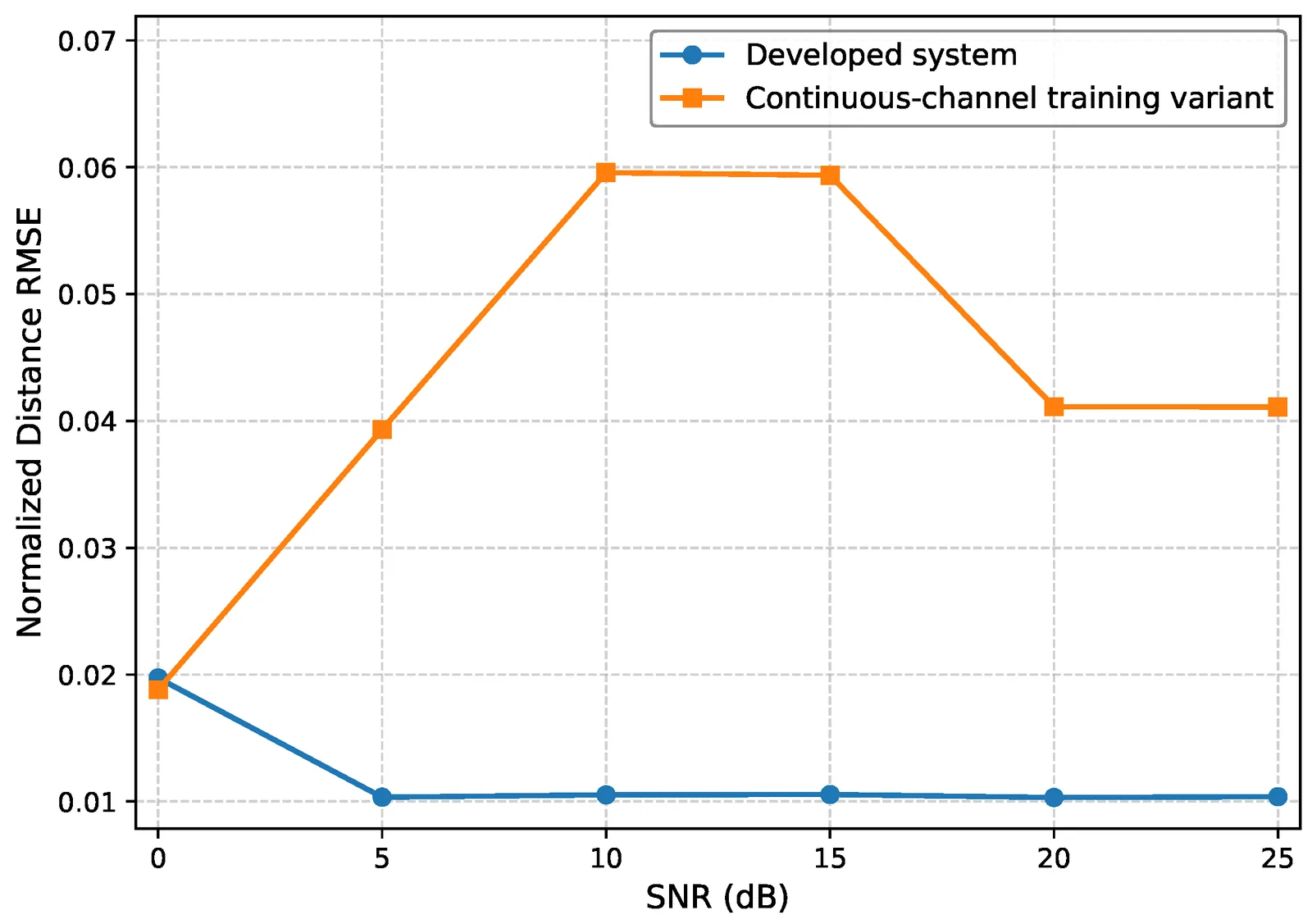
Normalized Distance RMSE under adaptive wireless operating points.

**Figure 6:**
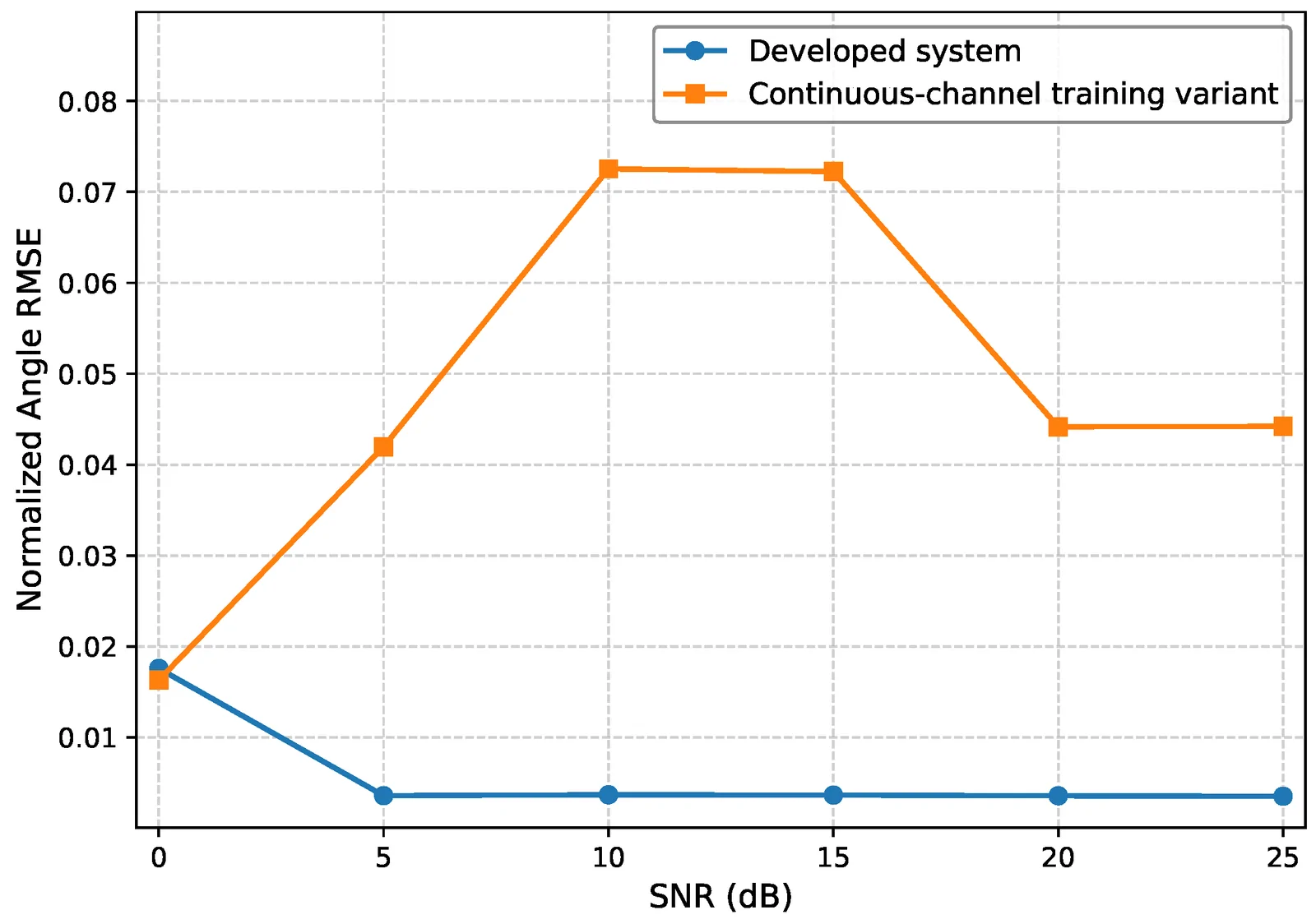
Normalized Angle RMSE under adaptive wireless operating points.

**Figure 7:**
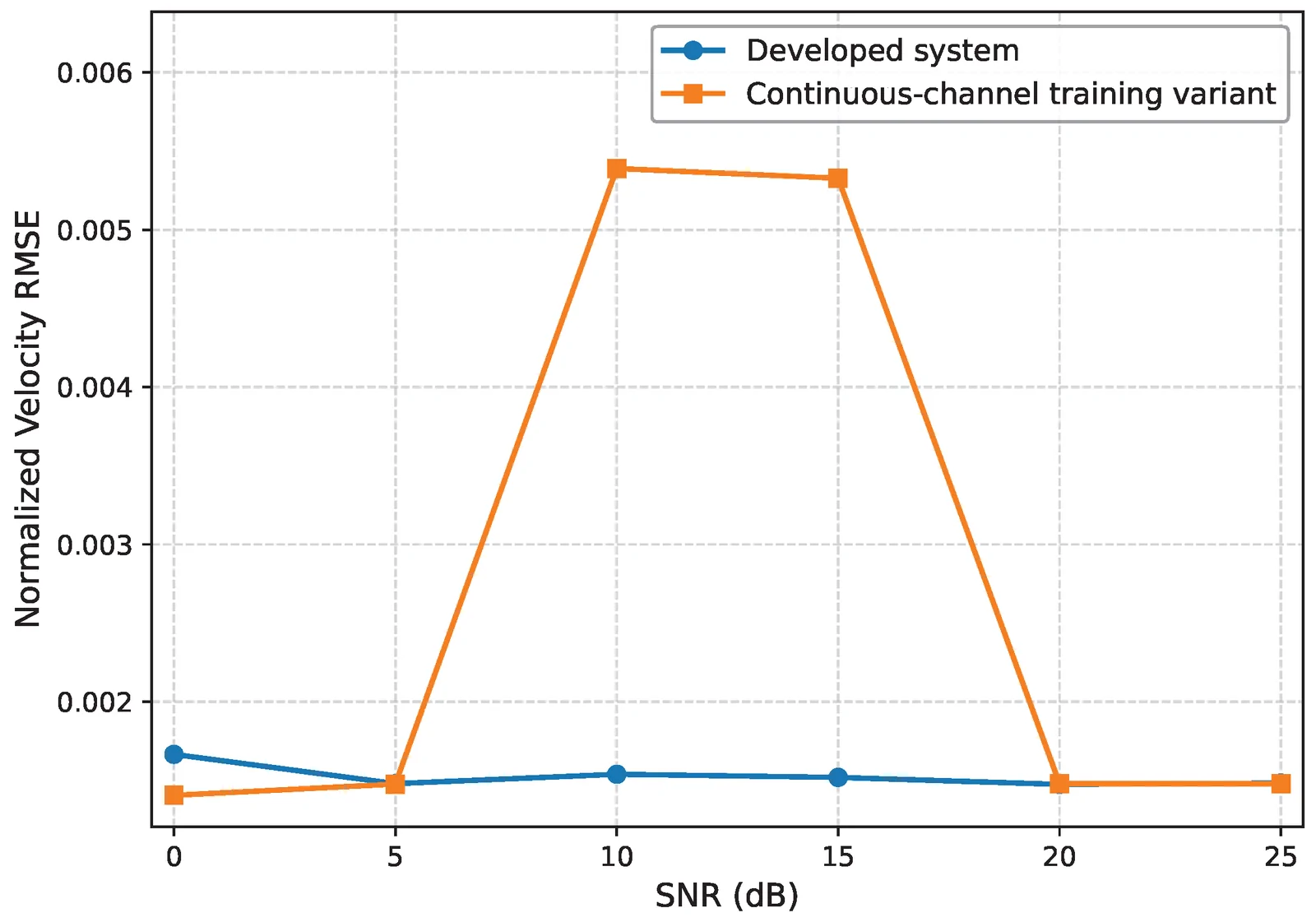
Normalized Velocity RMSE under adaptive wireless operating points.

The SNR-dependent performance is shown in Figure 3, Figure 4, Figure 5, Figure 6 and Figure 7. Figure 3 and Figure 4 report visual reconstruction quality, while Figure 5, Figure 6 and Figure 7 report normalized state-estimation accuracy. Across the evaluated adaptive wireless operating points, the developed system provides better visual reconstruction quality and lower normalized state-estimation errors than the continuous-channel training variant. This indicates that the learned vision–radar semantic representation remains effective after communication-aware semantic transmission.

### 5.3. Ablation on Modality Availability

This section evaluates the contribution of visual and radar modalities to the developed system. Table 3 compares the vision-only semantic transmission variant, the radar-only semantic transmission variant, and the developed vision–radar system under the same evaluation setting.

As shown in Table 3, the vision-only variant gives limited state-estimation accuracy because radar-related sensing cues are not available. The radar-only variant provides better normalized state estimation than the vision-only variant and also produces stronger visual reconstruction metrics, but it lacks the appearance information contained in the visual observation. The vision–radar semantic transmission setting achieves the best PSNR, SSIM, Normalized Distance RMSE, and Normalized Angle RMSE, showing that the two modalities provide complementary information for joint target image reconstruction and normalized state estimation. The Normalized Velocity RMSE is already very small for all compared modality settings, so the difference is less pronounced after rounding.

### 5.4. Ablation on Reconstruction Objectives

This section studies how different reconstruction objectives affect the visual-sensing performance of the developed system. Since the receiver is required to reconstruct the target image while also estimating normalized target states, the reconstruction objective may influence both visual quality and the learned multimodal semantic representation.

Table 4 compares three reconstruction objectives: MSE objective, MSE+L1 objective, and composite reconstruction objective. The MSE objective focuses on pixel-wise reconstruction fidelity. The MSE+L1 objective additionally introduces an absolute-error constraint. The composite reconstruction objective further incorporates structural reconstruction information and is adopted in the developed system.

As shown in Table 4, the composite reconstruction objective achieves the highest PSNR and SSIM, indicating improved target image reconstruction quality. It also obtains the lowest Normalized Distance RMSE and Normalized Angle RMSE. The displayed Normalized Velocity RMSE values remain close across the compared settings after rounding. Overall, the composite reconstruction objective provides the most favorable visual-sensing tradeoff for the developed system.

### 5.5. Ablation on Task-Balancing Schemes

This section studies the influence of different task-balancing schemes on the visual-sensing performance of the developed system. Since the receiver jointly reconstructs the target image and estimates the normalized target states, the relative balance among reconstruction and sensing objectives affects the learned multimodal semantic representation.

Table 5 compares four task-balancing schemes. The first three schemes adopt fixed task-balancing settings with different emphases, while the last scheme adopts the adaptive balancing strategy used in the developed system.

The mild fixed scheme achieves the highest SSIM, while the mild and state-aware fixed schemes give the lowest displayed Normalized Velocity RMSE after rounding. The state-aware fixed scheme obtains the highest PSNR. In contrast, the adaptive balancing scheme achieves the lowest Normalized Distance RMSE and Normalized Angle RMSE while maintaining visual reconstruction quality comparable to the fixed schemes.

These results indicate that different task-balancing schemes lead to different visual-sensing tradeoffs. The adaptive balancing scheme provides a favorable compromise for joint target-oriented recovery because it improves normalized distance and angle estimation without sacrificing visual reconstruction quality. The Normalized Velocity RMSE is already at a very small numerical level for all compared schemes, so the effect of changing the task-balancing strategy is less visible after rounding. From an optimization perspective, the adaptive balancing strategy mainly improves the tasks with larger remaining errors, especially normalized distance and angle estimation, while maintaining stable velocity-estimation performance.

### 5.6. Robustness Under Radar Perturbations

This section evaluates the robustness of the developed system under radar perturbation settings. The perturbations include additive complex Gaussian noise, random amplitude gain variation, and random phase rotation. These settings are used to examine the performance stability of the developed system during testing.

As shown in Table 6, radar perturbations lead to limited performance degradation in target image reconstruction and normalized state estimation. The developed system maintains stable PSNR, SSIM, and Normalized Distance RMSE, Normalized Angle RMSE, and Normalized Velocity RMSE under both low and high perturbation settings. This result indicates that the learned vision–radar semantic representation remains robust under the added radar perturbation settings.

### 5.7. Discussion

The experimental results demonstrate that the developed vision–radar semantic communication system can support target-oriented visual sensing by jointly reconstructing target images and estimating normalized dynamic states under adaptive wireless operating points. The representative reconstruction results show that the receiver can recover vehicle contours and target-level appearance information from the transmitted semantic representation. The performance comparison under adaptive wireless operating points further shows that incorporating the communication process during training improves both visual reconstruction quality and normalized state-estimation accuracy compared with the continuous-channel training variant.

The modality-availability ablation analyzes the roles of visual and radar inputs under the same experimental setting. The vision-only variant lacks radar-derived state cues and therefore produces much larger distance and angle errors. The radar-only variant provides stronger state estimation than the vision-only variant because the radar echo contains range-, angle-, and Doppler-related cues, but it does not provide the same visual appearance information as the visual modality. The developed vision–radar system combines the complementary information of the two modalities and achieves the most favorable overall performance.

The reconstruction-objective and task-balancing ablations further explain the influence of training design choices. The composite reconstruction objective improves visual reconstruction by combining pixel-level fidelity, absolute-error constraints, and structural consistency. The adaptive balancing scheme improves normalized distance and angle estimation while maintaining competitive visual reconstruction quality. The Normalized Velocity RMSE is already at a very small numerical level for all compared task-balancing schemes, so its variation is less pronounced after rounding.

The radar perturbation experiment further evaluates robustness under radar perturbation settings. The results show that the developed system maintains stable performance under low and high perturbation settings. This supports the robustness of the learned multimodal semantic representation when the radar input is affected by noise, amplitude variation, and phase rotation.

Overall, these findings show that the developed system can jointly recover target appearance and normalized dynamic state information under the evaluated vision–radar semantic communication setting.

## 6. Conclusions

Motivated by the research gap identified in the Introduction and Related Work, this paper developed a vision–radar semantic communication system for target-oriented visual sensing. Existing image-oriented semantic communication methods mainly focus on visual transmission or reconstruction, existing multimodal or multi-task semantic communication methods are generally not formulated for joint vision–radar target recovery, and radar–camera perception methods usually do not consider communication-aware semantic transmission. To address this gap, the developed system jointly encodes scene-level visual observations and target-associated radar echoes at the transmitter and recovers both the target image and normalized dynamic state information at the receiver.

The main contribution of this work is a unified vision–radar semantic communication framework that connects transmitter-side multimodal semantic extraction and fusion, lightweight contextual semantic encoding, communication-aware semantic transmission, and receiver-side multi-task semantic decoding. Within this framework, the receiver simultaneously reconstructs the target image and estimates normalized distance, angle, and velocity, enabling joint visual and state-oriented target recovery under adaptive wireless operating points.

Experimental results show that the developed system improves target image reconstruction quality and normalized state-estimation accuracy compared with the continuous-channel training variant. The modality-availability ablation further shows that vision–radar semantic transmission achieves better overall performance than the vision-only and radar-only variants, indicating the benefit of jointly exploiting visual and radar semantic information. The reconstruction-objective and task-balancing ablation studies show that the composite reconstruction objective and adaptive task-balancing strategy provide a favorable tradeoff between visual reconstruction and normalized state estimation. In addition, the radar perturbation analysis indicates that the developed system maintains stable performance under the added radar perturbation settings.

Future work will further investigate broader scene conditions, additional communication operating points, and the possible use of real measured radar data for further validation to extend the evaluation scope of vision–radar semantic communication.

## Figures and Tables

**Table 1:** Comparison of related studies and this work.

Category	Input	Semantic Trans.	Target Image Rec.	Target State Est.	Channel Model
Image semantic communication	Vision	Yes	Yes	No	Yes
Multimodal semantic communication	Multimodal	Yes	No	No	Yes
Multi-task semantic communication	Task-oriented	Yes	No	No	Yes
Radar–camera perception	Vision + radar	No	No	Yes	No
Developed system	Vision + radar	Yes	Yes	Yes	Yes

**Table 2:** Average performance of system variants under adaptive wireless operating points.

System Variant	PSNR ↑	SSIM ↑	Normalized Distance RMSE ↓	Normalized Angle RMSE ↓	Normalized Velocity RMSE ↓
Continuous-channel training variant	23.55	0.8670	0.0432	0.0486	0.0028
Developed system	**24.57**	**0.8896**	**0.0120**	**0.0059**	**0.0015**

**Table 3:** Average performance with different modality settings.

Modality Setting	PSNR ↑	SSIM ↑	Normalized Distance RMSE ↓	Normalized Angle RMSE ↓	Normalized Velocity RMSE ↓
Vision-only semantic transmission	12.41	0.5323	0.1985	0.0841	0.0015
Radar-only semantic transmission	20.67	0.8061	0.0266	0.0068	**0.0014**
Vision–radar semantic transmission (ours)	**24.57**	**0.8896**	**0.0120**	**0.0059**	0.0015

**Table 4:** Average performance with different reconstruction objectives.

Reconstruction Objective	PSNR ↑	SSIM ↑	Normalized Distance RMSE ↓	Normalized Angle RMSE ↓	Normalized Velocity RMSE ↓
MSE objective	24.29	0.8681	0.0129	0.0063	0.0015
MSE+L1 objective	23.01	0.8433	0.0226	0.0098	0.0015
Composite reconstruction objective (ours)	**24.57**	**0.8896**	**0.0120**	**0.0059**	0.0015

**Table 5:** Average performance with different task-balancing schemes.

Task-Balancing Scheme	PSNR ↑	SSIM ↑	Normalized Distance RMSE ↓	Normalized Angle RMSE ↓	Normalized Velocity RMSE ↓
Balanced fixed scheme	24.48	0.8883	0.0141	0.0065	0.0015
Mild fixed scheme	24.53	**0.8897**	0.0144	0.0066	**0.0014**
State-aware fixed scheme	**24.58**	0.8895	0.0131	0.0066	**0.0014**
Adaptive balancing scheme (ours)	24.57	0.8896	**0.0120**	**0.0059**	0.0015

**Table 6:** Average performance under radar perturbation settings.

Radar Setting	PSNR ↑	SSIM ↑	Normalized Distance RMSE ↓	Normalized Angle RMSE ↓	Normalized Velocity RMSE ↓
Unperturbed (ours)	**24.57**	**0.8896**	**0.0120**	**0.0059**	0.0015
Low perturbation	24.48	0.8873	0.0149	0.0065	0.0015
High perturbation	24.29	0.8830	0.0170	0.0071	0.0015

## Data Availability

The raw/processed data required to reproduce these findings cannot be shared at this time as the data also form part of an ongoing study.
